# Biochemical Analysis of Dimethyl Suberimidate-crosslinked Yeast Nucleosomes

**DOI:** 10.21769/BioProtoc.2770

**Published:** 2018-03-20

**Authors:** Yuichi Ichiakwa, Paul D. Kaufman

**Affiliations:** Department of Molecular, Cell, and Cancer Biology, University of Massachusetts Medical School, Worcester, MA, USA

**Keywords:** Chromatin, Nucleosome, Histone, Protein-protein interaction, Crosslink, Streptavidin affinity chromatography

## Abstract

Nucleosomes are the fundamental unit of eukaryotic chromosome packaging, comprised of 147 bp of DNA wrapped around two molecules of each of the core histone proteins H2A, H2B, H3, and H4. Nucleosomes are symmetrical, with one axis of symmetry centered on the homodimeric interaction between the C-termini of the H3 molecules. To explore the functional consequences of nucleosome symmetry, we designed an obligate pair of H3 heterodimers, termed H3X and H3Y, allowing us to compare cells with single or double H3 alterations. Our biochemical validation of the heterodimeric X-Y interaction included intra-nucleosomal H3 crosslinking using dimethyl suberimidate (DMS). Here, we provide a detailed protocol for the use of DMS to analyze yeast nucleosomes.

## Background

Post-translational modifications of histone proteins affect every aspect of chromosome biology, including transcription, replication, repair, and recombination. Because nucleosomes contain two copies of each core histone, modifications could be symmetric (same modifications on both H3 tails, *e.g.*, K27me on both H3 tails within a nucleosome (Voigt *et al.*, 2012)) or asymmetric (modifications on a single H3 tail, *e.g.*, K27me on a single H3 tail within a nucleosome (Voigt *et al.*, 2012)). Recent studies have demonstrated that nucleosomes in mammalian cells indeed display some asymmetric modifications (Voigt *et al.*, 2012; Shema *et al.*, 2016). To allow experimental manipulation of nucleosomal symmetry *in vivo*, we designed a pair of altered histone H3 proteins that have obligate heterodimeric interactions, termed H3X (L126A, L130V) and H3Y (L109I, A110W, L130I) (Ichikawa *et al.*, 2017). Yeast cells expressing both H3X and H3Y are viable, but inviable if cells express only H3X or H3Y.

**Figure 1.**
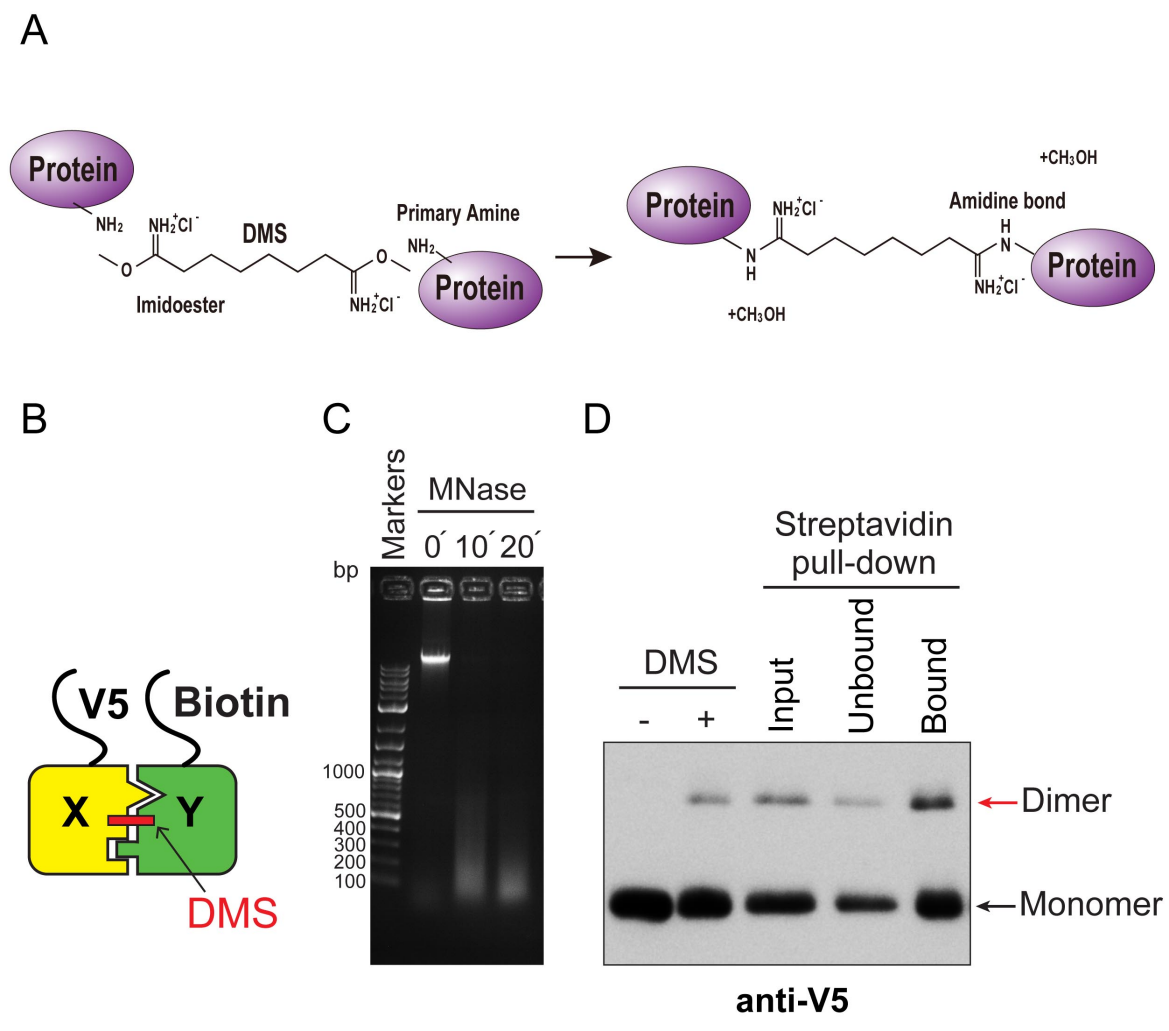
Biochemical validation of asymmetric nucleosome formation *in vivo*. A. Chemistry of DMS cross-linking. DMS reacts with primary amines of proteins to form amidine bonds. B. Schematic for DMS crosslink of H3X and H3Y heterodimer. Yeast strains expressed V5-tagged H3X and Biotin-tagged H3Y, as indicated. C. DNA samples purified from MNase-digested chromatin from each time point (0, 10, 20 min) were analyzed by electrophoresis on a 1.5% TAE agarose gel, and stained with ethidium bromide. Note that after DMS crosslinking, the MNase-digested DNA fragments do not display the characteristic polynucleosomal ladder of uncrosslinked chromatin. D. Immunoblot analysis of V5-H3X and biotin-H3Y interactions. The left two lanes show total uncrosslinked and DMS-crosslinked chromatin, and right lanes show MNase-digested chromatin (Input), flow through fraction (Unbound) and streptavidin-precipitated biotinylated-H3 (Bound). Samples were separated by 17% SDS-PAGE, transferred to a membrane, and probed with anti-V5 antibody.

For biochemical validation of H3X-H3Y interactions within individual nucleosomes, we generated yeast strains expressing the bacterial biotin ligase BirA, N-terminal V5-tagged H3X and N-terminal biotin-accepting epitope tagged H3Y (Beckett *et al.*, 1999). BirA is an enzyme that attaches biotin to a specific acceptor epitope, enabling us to purify the biotinylated molecules by streptavidin affinity chromatography. We treated extracts from yeast cells with dimethyl suberimidate (DMS), a crosslinking agent that contains a primary amine reactive imidoester group at each end of an 8-atom spacer arm (Figure 1A). DMS produces well-characterized crosslinks within histone octamers, including links between the two H3 molecules (Figure 1B; Kornberg and Thomas, 1974; Thomas, 1989). Therefore, this method can be used to report on the composition of asymmetric epitope tags.

Crosslinked samples are digested with micrococcal nuclease (MNase) to generate a soluble population of chromatin fragments containing approximately mononucleosome-sized DNA molecules (we note that DMS crosslinking prevents generation of a uniform ladder of MNase-digested products, Figure 1C). Biotin-tagged, MNase-digested chromatin is then purified via streptavidin-agarose affinity purification in the presence of high salt (2 M NaCl). This salt concentration is sufficient to remove DNA from histones (Bartley and Chalkley, 1972), avoiding interference from any neighbor nucleosomes that survived the MNase digestion. Bound proteins are then analyzed by Western blotting (Figure 1D). The DMS crosslinking efficiency of the X-Y heterodimeric pairs was around 10%, nearly identical to wild-type H3 homodimeric pairs; additionally, approximately 20% of the crosslinked heterodimers in the input fractions were precipitated by streptavidin-agarose (Ichikawa *et al.*, 2017). We applied this method to analyze the extent of homodimerization of H3X or H3Y, as well as X-Y heterodimerization (Ichikawa *et al.*, 2017). To examine this, we quantified X-Y dimer bands rather than the monomer, because these DMS crosslinked species represent direct H3-H3 interactions within individual nucleosomes.

## Materials and Reagents

1. 200 μl and 1,000 μl Pipette tips (Corning, Axygen^®^, catalog numbers: RFL-222-C, RFL-1200-C)
2. 1.5 ml O-ring screw-cap tubes (Fisher Scientific, catalog number: 02-707-353)
3. 1.5 ml microfuge tubes (Corning, Axygen^®^, catalog number: MCT-150-C)
4. 0.5 ml glass beads (Bio Spec Product, catalog number: 11079105)
5. 26 gauge needle (BD, catalog number: 305115)
6. 12 x 75 mm plastic tube (Corning, Falcon^®^, catalog number: 352008)
7. Nitrocellulose blotting membrane (GE Healthcare, catalog number: 10600004)
8. Examination gloves (Fisher Scientific, catalog number: 19-130-1597D)
9. Biotin (Sigma-Aldrich, catalog number: B4501)
10. Dimethyl suberimidate (DMS) (Thermo Fisher Scientific, Thermo Scientific^TM^, catalog number: 20700)
11. Trichloroacetic acid (TCA) (Fisher Scientific, catalog number: BP555)
12. Magnesium chloride hexahydrate (MgCl_2_·6H_2_O) (Acros Organics, catalog number: 197530010)
13. Calcium chloride dihydrate (CaCl_2_·2H_2_O) (Merck, Millipore Sigma, catalog number: 102382)
14. Disodium ethylenediamine tetraacetate (EDTA) (Fisher Scientific, catalog number: S311)
15. Ethylene glycol tetraacetic acid (EGTA) (Sigma-Aldrich, catalog number: E4378)
16. RNase A (Sigma-Aldrich, Roche Diagnostics, catalog number: 10109169001)
17. Proteinase K (Sigma-Aldrich, catalog number: P2308)
18. Ammonium acetate (NH_4_Ac) (Fisher Scientific, catalog number: A637)
19. Phenol:Chloroform:Isoamyl Alcohol (PCI) (Thermo Fisher Scientific, catalog number: 15593031)
20. 2-Propanol (Fisher Scientific, catalog number: A416)
21. Ethanol (Decon Labs, catalog number: 2701)
22. TE (10 mM Tris-Cl, 1 mM EDTA, pH 8.0).
23. 6x gel loading dye (New England Biolabs, catalog number: B7042)
24. Agarose (Fisher Scientific, catalog number: BP160)
25. Ethidium bromide (Sigma-Aldrich, catalog number: E7637)
26. CL2B Sepharose beads (Sigma-Aldrich, catalog number: CL2B300)
27. Streptavidin Sepharose beads (GE Healthcare, catalog number: 17-5113-01)
28. Insulin (Sigma-Aldrich, catalog number: I1882)
29. Clarity Western ECL Substrate (Bio-Rad Laboratories, catalog number: 1705060)
30. Primary antibody: anti-V5 tag (Thermo Fisher Scientific, Invitrogen, catalog number: R960-25)
31. Secondary antibody: anti-Mouse IgG (Thermo Fisher Scientific, Invitrogen, catalog number: 31430)
32. Nonfat dry milk (Walmart, Great Value)
33. Yeast nitrogen base without amino acids (United States Biological, catalog number: Y2025)
34. Glucose (Merck, Millipore Sigma, catalog number: DX0145)
35. Micrococcal Nuclease (MNase) (Worthington Biochemical, catalog number: LS004797)
36. Tris hydroxymethyl aminomethane (Tris) (Fisher Scientific, catalog number: BP152)
37. Sodium tetraborate decahydrate (Na borate) (Sigma-Aldrich, catalog number: S9640)
38. Sodium chloride (NaCl) (Fisher Scientific, catalog number: S271)
39. Hydrochloric acid (HCl) (Fisher Scientific, catalog number: A144)
40. Phenylmethylsulfonyl fluoride (PMSF) (RPI, catalog number: P20270)
41. Sodium dodecylsulfate (SDS) (RPI, catalog number: L22010)
42. Glycerol (Fisher Scientific, catalog number: G33)
43. Bromophenol blue (Sigma-Aldrich, catalog number: B7021)
44. 2-Mercaptoethanol (Sigma-Aldrich, catalog number: M3148)
45. Tween 20 (Sigma-Aldrich, catalog number: P2287)
46. Acetic acid, Glacial (Fisher Scientific, catalog number: A38)
47. 40% acrylamide solution (Bio-Rad Laboratories, catalog number: 1610140)
48. 2% Bis solution (Bio-Rad Laboratories, catalog number: 1610142)
49. Ammonium persulfate (Fisher Scientific, catalog number: BP179)
50. Tetramethylethylenediamine (TEMED) (Bio-Rad Laboratories, catalog number: 1610800)
51. Sodium bicarbonate (NaHCO_3_) (Fisher Scientific, catalog number: S233)
52. Sodium carbonate (Na_2_CO_3_) (Fisher Scientific, catalog number: S263)
53. Sodium hydroxide (NaOH) (Fisher Scientific, catalog number: S318)
54. Methanol (Fisher Scientific, catalog number: A412)
55. Synthetic media (see Recipes)
56. MNase (see Recipes)
57. Extraction (E) buffer (see Recipes)
58. 2x SDS-sample buffer (SB) (see Recipes)
59. Wash (W) buffer (see Recipes)
60. 17% SDS-PAGE gel (see Recipes)
61. 5% stacking gel (see Recipes)
62. 1x SDS-running buffer (see Recipes)
63. 40x Na carbonate buffer (see Recipes)
64. Blotting buffer (see Recipes)
65. TBST (Tris-buffered saline + Tween 20) (see Recipes)

## Equipment

1. P20, P200 and P1000 Pipettes (Gilson)
2. Labquake Tube Shaker/Rotators (Thermo Fisher Scientific)
3. Swing bucket rotor (Beckman Coulter, model: GH 3.8)
4. Shaker incubator (INFORS HT)
5. Mini-Beadbeater-96 (Bio Spec Product)
6. Tabletop centrifuge (Beckman Coulter, model: Allegra 6R)
7. Centrifuge for microcentrifuge tubes (Eppendorf, model: 5415 D)
8. ChemiDoc Touch Imaging System (Bio-Rad Laboratories, model: ChemiDoc Touch Imaging System)
9. Vortex-Genie 2 (Scientific Industries, model: Vortex-Genie 2)
10. Water bath (Precision Scientific, catalog number: 66551)
11. Vertical Mini-Gel systems (C.B.S. Scientific, model: MGV102)
12. Transfer electrophoresis unit (Hoefer, model: TE22)
13. EASY-CAST Electrophoresis System (Thermo Fisher Scientific, Thermo Scientific^TM^, model: Owl^TM^ Easy-Cast^TM^ B1)
14. Power Supply (Bio-Rad Laboratories, model: PowerPac^TM^ Basic)

## Software

1. Image Lab (Bio-Rad Laboratories)

## Procedure

**Day 1**

Pick a single colony from a plate, and inoculate an overnight culture of cells in 15 ml of synthetic media. Grow at 30 °C, 170 rpm in a shaker incubator. Do this 1-2 days beforehand, depending on growth rate of strain.

**Day 2**

Inoculate the overnight culture grown on day 1 into 110 ml of synthetic media supplemented with 250 nM biotin. Biotin is added to favor *in vivo* biotinylation of the tagged H3 proteins. The amount of cells to inoculate depends on growth rate (see below; typically, inoculate 3 OD units of cells of most X-Y strains into 110 ml media, which are then grown for 12 h). Grow at 30 °C, 170 rpm in a shaker incubator.

**Day3**

- Cross-linking H3 dimers with DMS
  1. Harvest at desired cell density. The desired cell density is 0.25 at OD_600_; don’t use more than 100 ml of cells at OD_600_ = 0.3 per Streptavidin-pull down described below, in order to assure that chromosomes are adequately digested with 20 μl MNase to generate mostly monosomes.
  2. Spin down cells in Swing bucket rotor at 2,000 *× g* for 10 min at 4 °C. Gently pour off supernatant.
  3. Resuspend cells in 0.5 ml E buffer at 4 °C and transfer to a 1.5 ml O-ring screw-cap tube. Spin down in microfuge 20 sec at max speed at 4 °C. Remove supernatant.
  4. Wash 3 times with 1 ml E buffer by vortex. Spin down in microfuge 20 sec at max speed at 4 °C. Remove supernatant. Thorough washing is important here to remove amine-containing compounds that will impair crosslinking.
  5. Resuspend each tube of cells completely in 900 μl E buffer at 4 °C, and add 0.5 ml glass beads.
  6. Bead beat: 3 times of 1 min beating (2,100 rpm) in the Mini-Beadbeater-96 (5 min between pulses, on ice).
  7. Heat a 26 gauge needle with a gas burner (Figure 2A), and puncture the tube bottom with the red-hot needle (Figure 2B). Place into a 12 x 75 mm plastic tube (‘FACS tube’) (Figure 2C) and spin for 2 min at 365 *× g* in a tabletop centrifuge at 4 °C (Figure 2D). Discard screwcap tube with glass beads (Figure 2E).
    **Figure 2.**
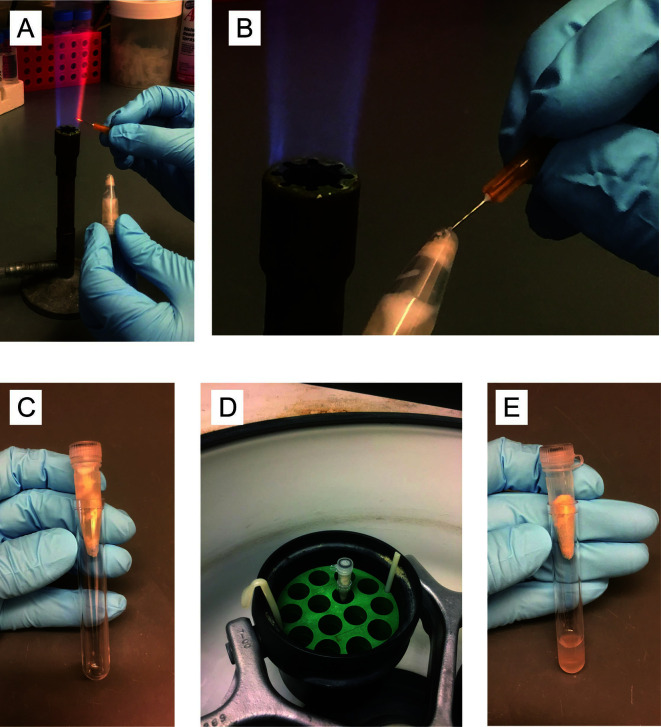
Step by step photos of the procedure Day 3, Step A7
  8. Resuspend pellet in the liquid (a mixture of E buffer and cell lysate on the bottom of FACS tube) completely, and transfer to a 1.5 ml microfuge tube.
  9. Make DMS stock 11 mg/ml in E buffer (typically 1 ml) at room temperature. This stock should be made freshly every time.
  10. Remove 0 min aliquot, 100 μl for SDS-PAGE. Add 1/10 volume 100% TCA. Incubate at room temperature for 10 min. Spin down in a microfuge for 10 min at max speed at room temperature. Remove supernatant, wash pellet with 1 ml of acetone at room temperature. Leave the lid open for 30 min to air-dry the pellet. Resuspend air dried pellet in 50 μl 2x SB. Store at -20 °C.
  11. Add 1/10 volume of 11 mg/ml DMS to a final concentration of 1 mg/ml. Incubate at room temperature with rotating for 60 min.
  12. Add 1/20 volume of 1 M Tris-HCl, pH 7.5 to a final concentration of 50 mM for quenching the DMS crosslinking and further rotation for 15 min at room temperature.
  13. Remove 60 min aliquot, 100 μl for SDS-PAGE. Add 1/10 volume 100% TCA to the aliquot, process as above.
  14. Go next step (MNase digestion) immediately after the cross-linking.
- MNase digestion
  1. Add 1/100 volume of 1 M MgCl_2_ to a final concentration of 10 mM (really 8 mM final, since E buffer contains 2 mM EDTA), and add 1/100 volume of 0.1 M CaCl_2_ to a final concentration of 1 mM to the DMS-crosslinked sample (the remaining amount after Step A-13, approximately 800 μl) at room temperature. Take 100 μl to generate an un-digested DNA sample for gel analysis. Store on ice.
  2. Equilibrate at 37 °C in a water bath for 5 min.
  3. Add 20 μl of MNase, invert the tubes 5 times and incubate at 37 °C for 20 min.
  4. Add 1/25 volume of 0.25 M EDTA/EGTA to a final concentration of 10 mM and invert 5-10 times to inhibit MNase. Take 100 μl to generate an MNase-digested DNA sample. Spin at 8,000 *× g* for 1 min, 4 °C, take supernatant for Streptavidin-pull down.
- Gel analysis of MNase digestion
  1. For DNA purification, add 5 μl of RNase A (10 mg/ml) to the 100 μl sample aliquots and incubate at 37 °C for 30 min.
  2. Add 5 μl of 20% SDS and 2 μl of Proteinase K (20 mg/ml). Incubate at 65 °C for 3 h.
  3. Add 200 μl of 7.5 M NH_4_Ac and 300 μl of ddH_2_O.
  4. Add 600 μl of PCI and vortex. Spin for 5 min in a microfuge at max speed.
  5. Take aqueous phase, and precipitate with 1 volume of 2-propanol.
  6. Spin down for 20 min at max speed at room temperature right after isopropanol precipitation.
  7. Wash pellet with 1 ml of 70% ethanol, spin for 5 min and air dry for 1 h.
  8. Resuspend pellet in 10 μl of TE.
  9. Add 2 μl of 6x loading buffer and run on 1.5% TAE agarose gel containing 0.5 μg/ml ethidium bromide at 5 V/cm for 50 min.
- Streptavidin-pull down
  1. Equilibrate CL2B Sepharose beads with E buffer at 4 °C. To block the beads, add 10 μg insulin per 40 μl slurry for each sample, rotate for 30 min at 4 °C. Add MNase digested samples (described at Step B4) to 40 μl slurry preblocked CL2B Sepharose beads, rotate for 30-60 min at 4 °C.
  2. Spin at 8,000 *× g* for 1 min, 4 °C. Take 30 μl supernatant + 30 μl 2x SB as ‘Input’ sample. Store at -20 °C.
  3. Transfer the supernatant into a new 1.5 ml tube. Add 30 μl slurry streptavidin-Sepharose beads preblocked with insulin (use 10 μg insulin per 30 μl slurry for each sample, process as above), rotate 2 h at 4 °C.
  4. Spin at 8,000 *× g* for 1 min, 4 °C. Take 30 μl supernatant + 30 μl 2x SB as ‘Unbound’ sample.
  5. Wash beads three times with 1 ml W buffer at 4 °C, by rotating at 4 °C for 5 min each time. Spin at 8,000 *× g* for 1 min at 4 °C, then discard supernatant.
  6. After the last wash, remove all supernatant with a 26 gauge needle on a vacuum. Resuspend the beads in 50 μl 2x SB. Store at -20 °C.
- Western blot
  1. Boil samples at 100 °C for 10 min.
  2. Load 10 μl of 0 min and 60 min samples, 15 μl of Input samples and 8 μl of Bound samples. Run on 17% SDS-PAGE gels in 1x SDS-running buffer at constant 8 mA while bromophenol blue goes through the stacking gel, and 18 mA as it goes through the resolving gel. Continue running for 15 min after bromophenol blue runs off the bottom to improve the resolution of histones. Total run time is approximately 2 h.
  3. Transfer to Nitrocellulose membrane in blotting buffer at constant 0.5 A for 48 min.
  4. Block the membrane in 5% milk/TBST for 1 h at room temperature.
  5. Incubate membrane overnight at 4 °C with anti-V5 antibody (1:10,000 dilution in 5% milk/TBST).
  6. Wash the membrane three times each for 10 min with 5% milk/TBST.
  7. Incubate the membrane with secondary antibody (anti-Mouse IgG 1:15,000 dilution in 5% milk/TBST) at room temperature for 1 h.
  8. Wash the membrane three times each for 10 min with TBST.
  9. Remove the TBST and incubate the membrane with 1 ml ECL (enhanced chemiluminescence) solution for 10 min. Detect by using ChemiDoc Touch Imaging System (Chemiluminescent Blot mode, at the highest resolution).

## Data analysis

**Figure 3.**
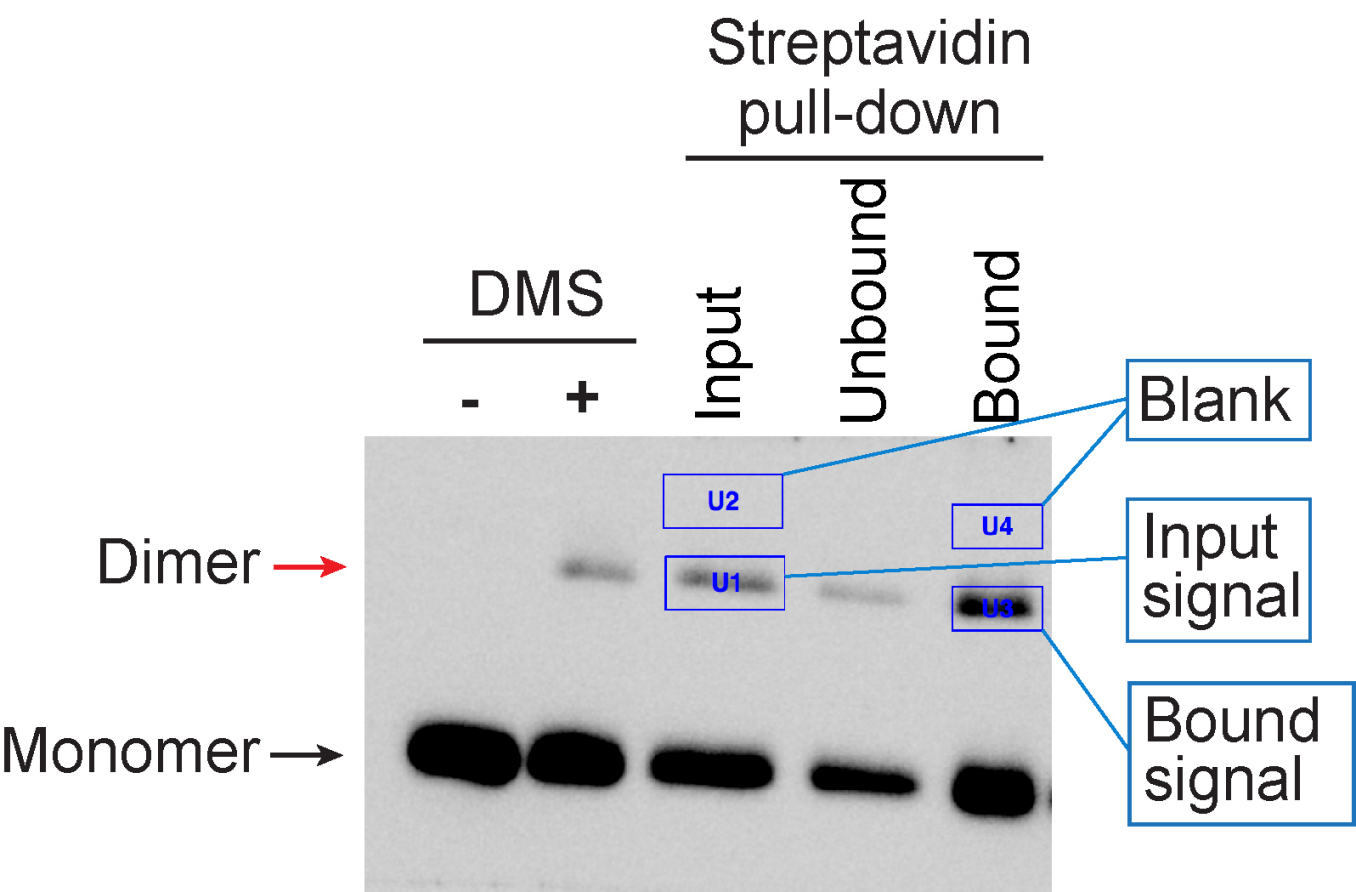
Data analysis with Bio-Rad image Lab. Areas of the H3-H3 dimer bands and the blanks were defined by surrounding it with a rectangle box using the ‘Volume Tools’.

H3-H3 crosslinked species were quantified with Bio-Rad ‘Image Lab’ software, using the ‘Volume Tools’. The area of the band was defined by surrounding it with a rectangle box (Figure 3). The same volume area was used to measure background signals, which were subtracted from the band intensity. The percentage of precipitated H3 dimer was calculated with following formulas:

Total input = ‘Band intensity of H3 dimer on input lane’ x ‘Total volume of input fraction’/‘Loading volume of input fraction’

Total bound = ‘Band intensity of H3 dimer on bound lane’ x ‘Total volume of bound fraction’/‘Loading volume of bound fraction’

Percentage of precipitated H3 dimer = (Total bound/Total input) x 100

The mean and standard deviation were calculated from the values of 3 independent replicate experiments.

## Notes

1. Use fresh cells (don’t freeze cells before the crosslink).
2. Use fresh DMS stock.
3. Note that during bead beating, visible aggregates appear, and many of these remain in the screwcap tube with the discarded glass beads at Step A7 of the DMS cross-link section. This is normal and does not indicate a problem.
4. 60 min of incubation during the DMS crosslinking reaction at Step A11 was sufficient for us to obtain crosslinked H3 dimers. However, you can change the incubation time depending on your purpose and sample conditions.
5. PVDF membranes can be used for the Western blot, but in our experience Nitrocellulose membranes detect histones more sensitively.
6. Acrylamide is toxic. Wear examination gloves when you make SDS-PAGE gels.

## Recipes

1. Synthetic media
  0.67% yeast nitrogen base without amino acids
  2% glucose
*Note: No pH adjustment.*
2. MNase
  20 U/μl in 10 mM Tris-HCl pH 7.4
3. Extraction (E) buffer
  20 mM Na borate
  0.35 M NaCl
  2 mM EDTA
  Adjust pH to 9.00 with HCl
  1 mM PMSF (added freshly)
4. 2x SDS-sample buffer (SB)
  0.1 M Tris-HCl pH 6.8
  2% SDS
  20% glycerol
  0.02% bromophenol blue
  1/50 volume of 2-mercaptoethanol
5. Wash (W) buffer
  10 mM Tris-HCl pH 8.0
  1 mM EDTA
  2 M NaCl
  0.2% Tween 20
6. 17% SDS-PAGE gel (0.75 mm thickness, 14-well comb)
  2.13 ml of 40% acrylamide
  0.18 ml of 2% bisacrylamide
  1.875 ml of 1.0 M Tris-HCl pH 8.8
  25 μl of 20% SDS
0.78 ml of H_2_O
  20 μl of 10% ammonium persulfate
  5 μl of TEMED
7. 5% stacking gel
  0.31 ml of 40% acrylamide
  0.18 ml of 2% bisacrylamide
  0.31 ml of 1.0 M Tris-HCl pH 6.8
  12.5 μl of 20% SDS
1.69 ml of H_2_O
  10 μl of 10% ammonium persulfate
  5 μl of TEMED
8. 1x SDS-running buffer
  25 mM Tris
  192 mM glycine
  0.1% SDS
9. 40x Na carbonate buffer
251 mM NaHCO_3_
173 mM Na_2_CO_3_
  Adjust pH to 9.5 with NaOH
10. Blotting buffer
  1x Na carbonate buffer
  20% methanol
11. TBST
  25 mM Tris-HCl pH 8.0
  137 mM NaCl
  2.68 mM KCl
  0.1% Tween 20
